# Archaeal communities as indicators of hydrothermal influence in the Tianxiu vent field, Northwest Indian Ocean

**DOI:** 10.3389/fmicb.2026.1837947

**Published:** 2026-05-08

**Authors:** Fangru Li, Xiaolei Liu, Weiguo Hou, Hailiang Dong, Jinglong Hu, Hongyu Chen, Yingwen Zhong, Yuehong Wu, Xuewei Xu, Yi Ding

**Affiliations:** 1 State Key Laboratory of Geomicrobiology and Environmental Changes, China University of Geosciences, Beijing, China; 2 Frontiers Science Center for Deep-time Digital Earth, China University of Geosciences, Beijing, China; 3 State Key Laboratory of Submarine Geoscience, Second Institute of Oceanography, Ministry of Natural Resources, Hangzhou, China

**Keywords:** archaeal community composition, co-occurrence network, environmental filtering, hydrothermal gradient, *Hydrothermarchaeia*

## Abstract

Deep-sea hydrothermal sediments represent critical zones for archaea-driven biogeochemical cycling, yet the ecological differentiation of archaeal communities across hydrothermal gradients remains poorly understood. Here, we used 16S rRNA gene amplicon sequencing of sediment cores from two contrasting sites in the Tianxiu hydrothermal field of the Northwest Indian Ocean, and performed metagenomic analysis on the near-vent BC12 sediments, to investigate archaeal community composition, co-occurrence patterns, and metabolic potential in response to the hydrothermal activity. Comparative analysis revealed marked divergence between near-vent site BC12 and far-vent site JL218P. The site BC12, under stronger hydrothermal influence, was enriched in *Hydrothermarchaeia*, along with *Nanoarchaeia* and *Thermoplasmata*, and exhibited a more complex, highly connected co-occurrence network. Correlation analyses further showed that *Hydrothermarchaeia* abundance was significantly associated with hydrothermal-related geochemical gradients, supporting this lineage as a potential indicator of hydrothermal influence. Metagenomic analysis of BC12 further revealed *Hydrothermarchaeia* genomes encoding the Wood-Ljungdahl carbon fixation pathway, while genome-centric functional inference suggested enhanced potential for methanogenesis and hydrogen oxidation. In contrast, JL218P was dominated by *Nitrososphaeria*, showed limited vertical variation, and formed a simpler network structure, with predicted functional profiles more closely associated with nitrification and aerobic ammonia oxidation. Together, these findings identify hydrothermal-related geochemical heterogeneity as a major driver of archaeal community composition, ecological organization, and metabolic differentiation in deep-sea sediments, and advance our understanding of the ecological drivers structuring deep-sea hydrothermal ecosystems.

## 1 Introduction

Deep-sea hydrothermal vent systems, characterized by their extreme physical and chemical gradients, are among the most biologically productive and geochemically dynamic environments on Earth (Grassle, 1987; Sander and Koschinsky, 2011). These systems host unique microbial communities that drive fundamental biogeochemical cycles, independent of solar energy (Dick, 2019; Zeng et al., 2021). Archaea have emerged as key mediators in these processes, with distinct lineages occupying specialized metabolic niches shaped by local physicochemical gradients (Takai and Nakamura, 2011; Baker et al., 2020). This functional specialization is profoundly influenced by the local geologic setting. For instance, whereas *Thermococcales* species dominate high-temperature zones through sulfur respiration (Gorlas et al., 2022; Jiao et al., 2025), ANME groups facilitate methane cycling in anoxic niches (Teske et al., 2002), and *Nitrososphaeria* (synonym *Thaumarchaeota*) thrive in diffuse-flow environments through ammonia oxidation and carbon fixation (Luo et al., 2024; Zou et al., 2025). Recent studies further reveal novel lineages like *Hydrothermarchaeota* (formerly Marine Benthic Group E, MBG-E) as another important dominant microorganism in hydrothermal vent environments, which potentially facilitates dark hydrogen and carbon monoxide cycling in subsurface sediments (Carr et al., 2019; Zhou et al., 2020). Furthermore, investigations into hydrothermal systems have significantly broadened our understanding of microbial diversity and functional potential. Investigations of unique settings like the sediment-rich Guaymas Basin demonstrate how distinct geochemical contexts fundamentally shape microbial community structure and function (Ramírez et al., 2020). Ultramafic-hosted systems, like the Tianxiu hydrothermal field on the Carlsberg Ridge, are of particular interest due to serpentinization processes that generate fluids enriched in hydrogen and methane, creating thermodynamic landscapes that theoretically favor hydrogenotrophic methanogenesis and sulfur reduction (Schmidt et al., 2007; Schrenk et al., 2013).

The Tianxiu vent field represented the first active ultramafic-hosted hydrothermal system identified on the slow-spreading Carlsberg Ridge in the Northwest Indian Ocean (Han et al., 2015). Previous studies have confirmed significant geochemical heterogeneity in its surface sediments (Li et al., 2023; Qiu et al., 2023; Zhou et al., 2023). A recent study of the total microbial community further showed that microbial community structure shifts along the hydrothermal gradient (Li et al., 2025), supporting the idea that hydrothermal energy availability acts as an important environmental filtering in this system. However, that study has focused on broad patterns of the whole microbial assemblage and did not resolve archaeal-specific community composition, ecological organization, or metabolic differentiation. Given the central role of archaea in deep-sea biogeochemical cycling, their responses to hydrothermal forcing require targeted investigation.

To address these gaps and clarify the novelty relative to our previous whole-community analysis, we performed an archaeal-focused study of sediment cores collected along a pronounced hydrothermal gradient at the Tianxiu vent field, encompassing a near-vent (BC12) site and a far-vent (JL218P) site. By combining 16S rRNA gene amplicon sequencing and metagenomic analysis, we aimed to: (1) characterize archaeal community composition, (2) compare archaeal co-occurrence patterns under contrasting hydrothermal influence, and (3) assess archaeal ecological and metabolic differentiation, particularly with respect to carbon, sulfur, and nitrogen cycling. In this way, the present study moves beyond the previous whole-community survey and provides a domain-specific understanding of archaeal assembly, interactions, and functional potential along the hydrothermal gradient.

## 2 Materials and methods

### 2.1 Study sites and sampling

Sediment cores were collected from the Tianxiu hydrothermal vent field (TVF; 3°45′N, 63°45′E, ∼3,500 meters water depth), an active ultramafic-hosted system situated on the Carlsberg Ridge, Northwest Indian Ocean, during the R/V Dayang 72 cruise in 2022. Two cores, BC12 (3°41′30.822″N, 63°49′54.354″E, ∼3,400 meters water depth) and JL218P (3°41′43.955″N, 63°50′0.452″E, ∼3524.1 meters water depth) were selected for the present study. These cores were located approximately 40 meters and 500 meters from active hydrothermal vents, respectively, and were used to represent sediments subjected to different degrees of hydrothermal influence (Supplementary Figure S1). The cores and sediment subsamples analyzed here belong to the same sample set previously reported (Li et al., 2025), in which the cruise sampling procedures were described in detail. Briefly, following the sampling and subsampling procedures detailed therein, a total of 18 depth-stratified sediment samples were obtained and stored at −80 °C until downstream analyses. Geochemical profiles for both cores, including pH, redox potential (ORP), H_2_S, total carbon (TC), and metal-associated parameters, were used to characterize sediment geochemical conditions in the present study (Supplementary Figure S2).

### 2.2 DNA extraction, PCR amplification, and sequencing

Total genomic DNA was extracted from sediment subsamples of cores BC12 and JL218P using the DNeasy PowerSoil Pro Kit (QIAGEN, Germany) following the manufacturer’s protocol. DNA quantity and purity were evaluated using the Qubit fluorometer and the Nanodrop 1000 spectrophotometer, respectively (Thermo Fisher Scientific, USA). For archaeal community analysis, the V4-V5 regions of the 16S rRNA genes were amplified with the barcoded archaea-specific primer pair Arch519F (5′-CAGCCGCCGCGGTAA-3′) and Arch915R (5′-GTGCTCCCCCGCCAATTCCT-3′) as described (Wei et al., 2019). Each 50 μL PCR reaction mixture contained 50 ng of template DNA, 1 μL of each primer (10 μM), 1 μL BSA, 25 μL Taq polymerase Mix, and nuclease-free water. Amplification was performed under the following conditions: initial denaturation at 95 °C for 1 min, followed by 32 cycles of denaturation at 95 °C for 15 s, annealing at 53 °C for 15 s, and extension at 72 °C for 15 s, with a final extension at 72 °C for 5 min. The resulting amplicons were purified and sequenced on an Illumina Novaseq platform (PE250) by Magigene Biotechnology Co., Ltd. (Guangdong, China).

### 2.3 Bioinformatic analyses of 16S rRNA gene amplicons

Raw sequencing data were processed with VSEARCH (Rognes et al., 2016) for primer trimming, quality filtering, dereplication, and exclusion of low-abundance and chimeric sequences. The resulting high-quality sequences were clustered into operational taxonomic units (OTUs) at a 97% similarity threshold. Taxonomic assignment of OTU representatives was performed on the Galaxy platform (Feng et al., 2017) using the RDP classifier (Wang et al., 2007) with the SILVA database (v138.1) (Pruesse et al., 2007). To normalize uneven sequencing depth across samples, the OTU table was rarefied to 87,000 sequences per sample before downstream analyses.

Alpha diversity of the archaeal community was evaluated using the observed richness, Shannon index, Pielou’s index, and Faith’s phylogenetic diversity (PD) indices. Community compositional differences were assessed using Bray–Curtis dissimilarity and visualized by principal coordinates analysis (PCoA) in the vegan package (Oksanen et al., 2015) in R. Taxa contributing most to compositional dissimilarity between groups were identified using similarity percentage analysis (SIMPER) via PAST (v5.0.1) (Hammer et al., 2001). Relationships among geochemical variation, archaeal community composition, and specific taxon distributions were evaluated using Mantel tests, pairwise Spearman correlations, and principal component analysis (PCA). Potential functional profiles were predicted from the 16S rRNA gene sequences via Functional Annotation of Prokaryotic Taxa (FAPROTAX) (Louca et al., 2016), with particular focus on biogeochemical pathways associated with carbon, sulfur, and nitrogen cycling. Differences between the two core sediment communities were evaluated by *t*-test in the Statistical Analysis of Metagenomic Profiles (STAMP), with *p* < 0.05 considered statistically significant.

### 2.4 Metagenomic sequencing and analysis

Seven stratified sediment samples from BC12 (BC1201 to BC1207) were subjected to metagenomic sequencing on the NovaSeq™ X Plus platform at Novogene (Tianjin, China). Raw sequencing reads were processed following the MetaWRAP pipeline (Uritskiy et al., 2018). Initially, quality control was performed using the *read_qc* module to obtain high-quality clean reads. The quality-filtered reads were then assembled into contigs using MEGAHIT (Li et al., 2015). Genomic binning was implemented using MetaBAT2 (Kang et al., 2015), MaxBin2 (Wu et al., 2016), and CONCOCT (Alneberg et al., 2014). Resulting metagenome-assembled genomes (MAGs) from the different binning methods were integrated and refined using the *bin_refinement* module. MAG quality was assessed by CheckM (Parks et al., 2015), retaining bins with estimated completeness greater than 70% and contamination below 10%. Bin abundance was quantified with Salmon (Patro et al., 2017), and MAGs were further improved through reassembly using the *reassemble_bins* module. Taxonomic classification of refined MAGs was carried out using GTDB-Tk (Chaumeil et al., 2019). Coding sequences in the refined MAGs were predicted and annotated using Prokka (Seemann, 2014), and the resulting protein sequences were further searched against multiple specialized databases using DIAMOND, including the Kyoto Encyclopedia of Genes and Genomes (KEGG) (Kanehisa and Goto, 2000), NCycDB (Tu et al., 2019), SCycDB (Yu et al., 2021), and MCycDB (Qian et al., 2022).

To assess the consistency between archaeal functional predictions inferred from amplicon-based FAPROTAX analysis and metagenomic evidence in BC12, a targeted archaeal-focused analysis was performed on the assembled metagenomic contigs. Protein-coding genes were predicted from contigs using prodigal in metagenomic mode, and the predicted proteins were searched against a RefSeq archaeal protein reference database (O'Leary et al., 2016). Proteins with significant archaeal hits were retained as archaeal-affiliated proteins and subsequently annotated by searching against the KEGG database using DIAMOND blastp. Marker genes corresponding to archaeal functions predicted by FAPROTAX were then screened in the metagenome to evaluate concordance between the two approaches. Heatmaps were generated in R with the *pheatmap* package using row-scaled relative abundance values.

The temperature optima for microorganisms were generated from both the complete metagenomic contig set and individual MAGs, and analyzed using TOME (Li et al., 2019). Predictions derived from the complete metagenomic contig set were interpreted as proxies for community-level thermal adaptation, reflecting the aggregate thermal adaptation signal of the assembled metagenome. In parallel, predicted optimal growth temperatures were calculated for individual MAGs and used to assess genome-level thermal adaptation in specific archaeal lineages.

### 2.5 Co-occurrence network analysis

Archaeal co-occurrence networks across sediment cores were inferred based on the random matrix theory (RMT) using the iNAP Pipeline (Feng et al., 2022). OTUs present in more than 50% of the subsamples were retained for downstream network analysis. Pairwise associations among OTUs were estimated using Spearman’s rank correlation, and strong associations with a similarity threshold of 0.90 were retained as network edges. The network was visualized using the Fruchterman–Reingold algorithm in Gephi (0.10.1) (Bastian et al., 2009) and the modular grouping layout in Cytoscape (3.10.3) (Shannon et al., 2003).

To delineate topological differences between the networks, we quantified key global properties: the average degree (avgK), density (D), average path length (GD), harmonic geodesic distance (HD), average clustering coefficient (avgCC), centralization of betweenness (CB), connectedness (Con), and modularity (Feng et al., 2017). Keystone taxa were identified based on within-module connectivity (Zi) and among-module connectivity (Pi), which reflect a node’s connectivity within its own module and across different modules, respectively. Following Deng et al. (2012), nodes were classified as peripherals (Zi < 2.5, Pi < 0.62), connectors (Zi < 2.5, Pi > 0.62), module hubs (Zi ≥ 2.5, Pi < 0.62), and network hubs (Zi ≥ 2.5, Pi ≥ 0.62). Nodes in the latter three categories were considered keystone species, critical for maintaining network stability.

## 3 Results

### 3.1 Taxonomic composition in archaeal communities across sediment cores

**Figure 1:**
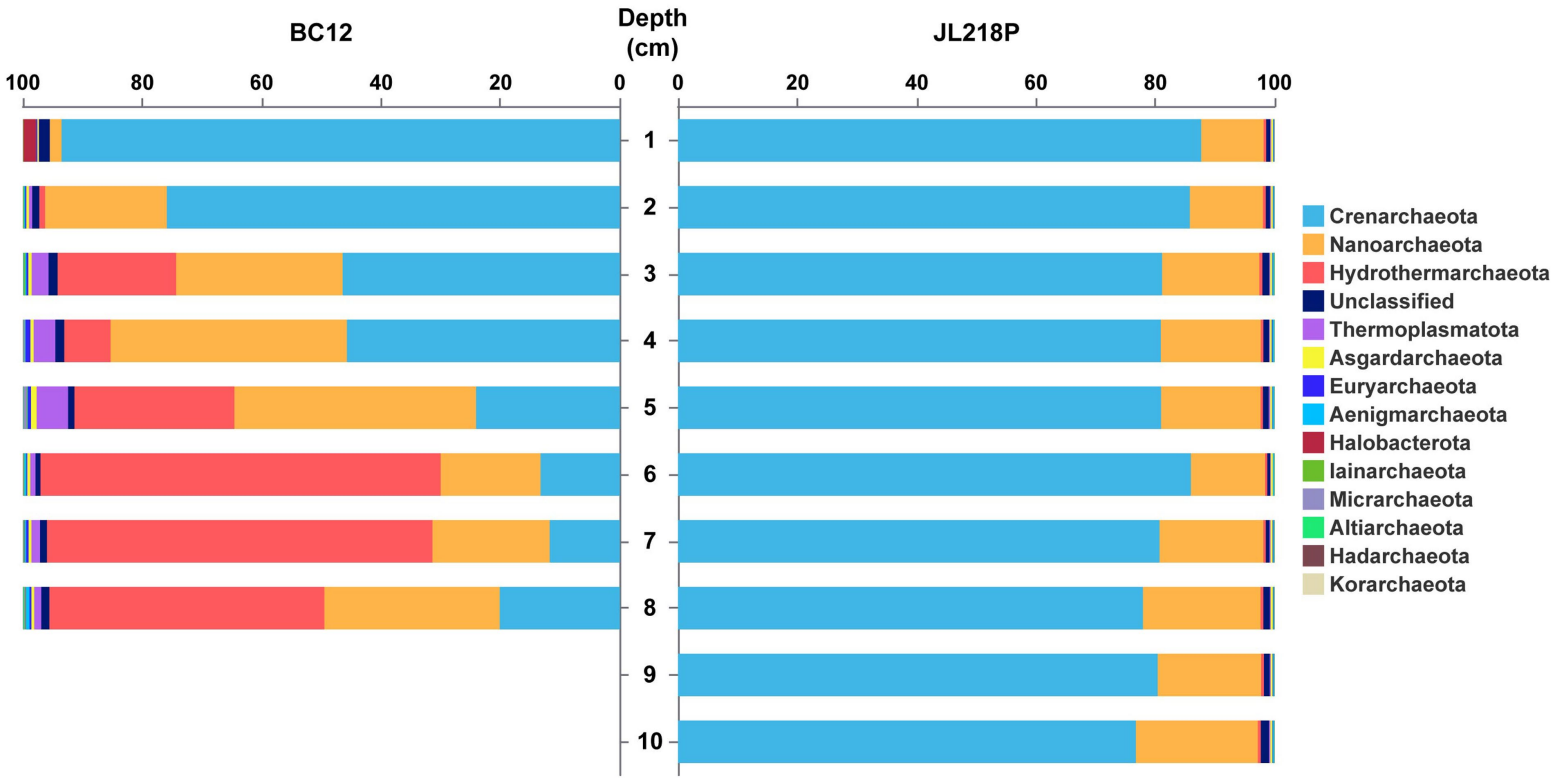
Relative abundance of the microbial community at the phylum level.

Archaeal community composition differed substantially between BC12 and JL218P, with stronger vertical variation observed in BC12. At the phylum level, BC12 was mainly dominated by *Crenarchaeota* (ranging from 11.75 to 93.55%), *Hydrothermarchaeota* (0.01–67.07%), and *Nanoarchaeota* (1.96–40.49%), whereas *Thermoplasmatota*, *Asgardarchaeota*, and *Euryarchaeota* were consistently minor components (Figure 1). Along the BC12 depth profile, the relative abundance of *Crenarchaeota* decreased progressively, while *Hydrothermarchaeota* increased and peaked at 6-cm depth (~67.07%; *p* < 0.05). *Nanoarchaeota* and *Thermoplasmatota* showed modest, non-significant increases in the 3–5 cm interval (*p* > 0.05).

In JL218P, the archaeal assemblage was comparatively uniform, dominated throughout by *Crenarchaeota* (76.74–87.69%) and *Nanoarchaeota* (10.48–20.45%). In contrast to BC12, *Hydrothermarchaeota* remained at very low abundance across all layers (0.36–0.52%) (Figure 1).

Consistent with these phylum-level patterns, SIMPER analysis at the class level showed that *Nitrososphaeria*, *Hydrothermarchaeia*, *Nanoarchaeia*, and *Thermoplasmata* accounted for most of the dissimilarity between the two sites (Table 1). *Nitrososphaeria* contributed nearly half of the total dissimilarity (49.29%) and was more abundant in JL218P (*p* < 0.05), whereas *Hydrothermarchaeia* (32.55% contribution), *Nanoarchaeia* (13.86%), and *Thermoplasmata* (2.05%) were all more enriched in BC12 (*p* < 0.05). Thus, between-site differences in archaeal community composition were largely explained by shifts in a small number of dominant taxa.

**Table 1:** Results of SIMPER analysis.

Taxa	Av. Diss	Contrib%	Cum%	BC12	JL218P
*Nitrososphaeria*	21.81	49.29	49.29	40.6	81.3
*Hydrothermarchaeia*	14.4	32.55	81.84	29.1	0.445
*Nanoarchaeia*	6.132	13.86	95.69	24.5	16
*Thermoplasmata*	0.906	2.047	97.74	1.94	0.163
*Unclassified*	0.3753	0.8482	98.59	1.61	0.912
*Bathyarchaeia*	0.1336	0.302	98.89	0.75	0.498
*Thermococci*	0.1025	0.2317	99.12	0.261	0.0702
*Lokiarchaeia*	0.1021	0.2308	99.35	0.51	0.325
Deep Sea Euryarchaeotic Group	0.06741	0.1523	99.51	0.168	0.123

Av, average cover percentage; Av. Diss, average dissimilarity; Contrib%, contribution (%); Cum%, Cumulative contribution (%).

### 3.2 Archaeal diversity and community structure of the two sediment cores

**Figure 2:**
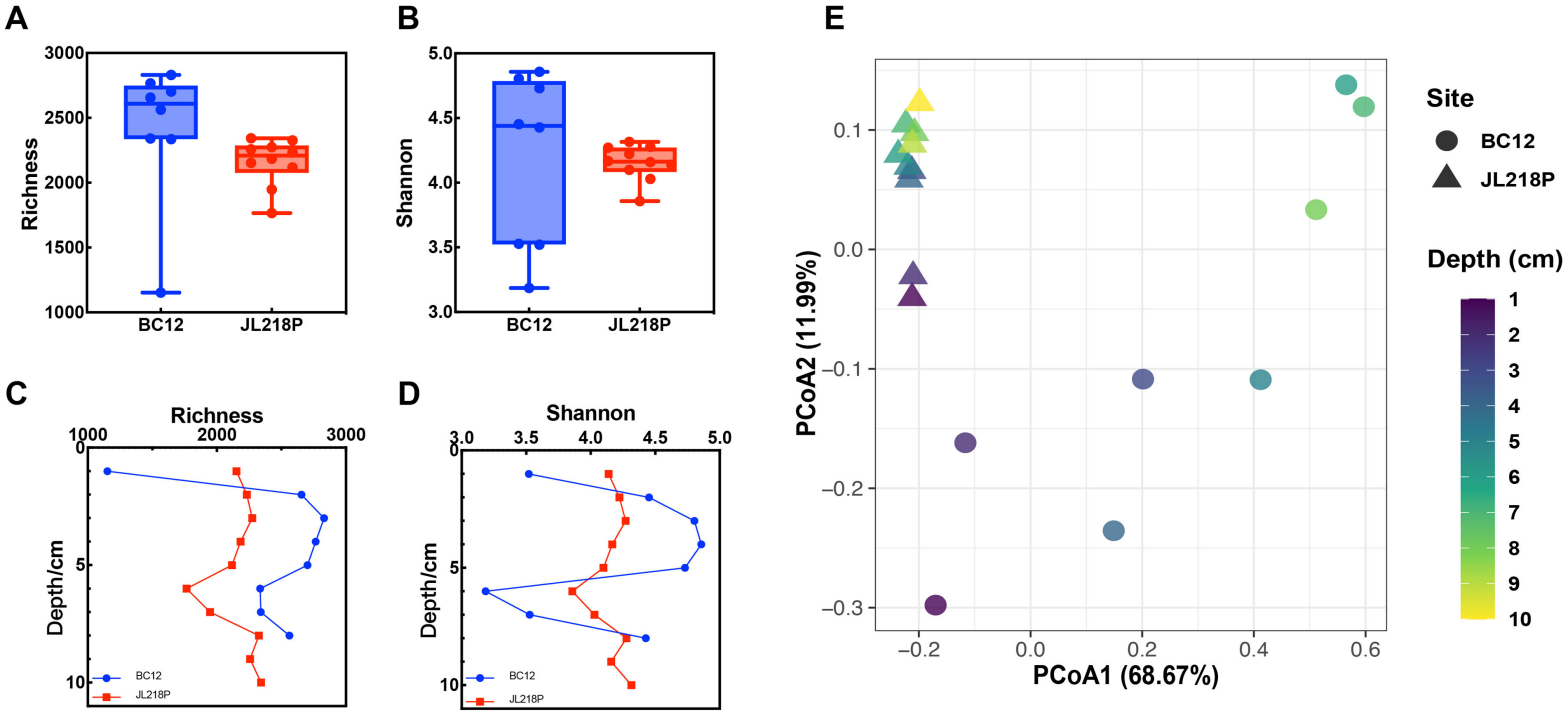
Diversity and community structure of the microbial community in the two sediment cores. **(A,B)** Box plots comparing the two sediment cores; **(C,D)** vertical profiles of alpha diversity index with depth. **(E)** PCoA analysis based on Bray–Curtis dissimilarity at OTU level.

Comparative analysis of archaeal alpha diversity showed that the near-vent BC12 consistently exhibited higher Richness and Shannon index values than the distal core JL218P, although these differences were not statistically significant (*p* > 0.05) (Figures 2A,B). Depth-resolved profiles further highlighted a pronounced contrast between the two sites. The BC12, both species richness and phylogenetic diversity decreased markedly below 5 cm, indicating a more dynamic diversity pattern. By contrast, JL218P displayed only limited variations along the core, with a relatively modest decline in deeper layers (5–8 cm) (Figures 2C,D). These observations indicate stronger vertical stratification of archaeal diversity in the near-vent sediments than in the far-vent sediments.

PCoA analysis based on beta diversity revealed a clear separation between archaeal communities from BC12 and JL218P (Figure 2E). The first two principal coordinates accounted for 80.66% of the total variation, with PCo1 and PCo2 explaining 68.67 and 11.99%. This pattern was supported by PERMANOVA, which detected a significant difference in community structure between the two cores (*p* < 0.001). Together, these results suggest that archaeal assemblages differed substantially between the near-vent and far-vent sediments, consistent with strong site-dependent environmental filtering.

### 3.3 Geochemical variation and its association with archaeal community structure

Geochemical parameters differed substantially between BC12 and JL218P and also varied with sediment depth (Supplementary Figure S2). In particular, H_2_S, TC, pH, ORP, and several metal-associated variables showed pronounced site- and depth-dependent patterns, indicating distinct sediment geochemical conditions between the two cores. Consistent with these environmental differences, statistical analyses revealed significant associations between geochemical variation and archaeal community composition.

The temperature was not measured *in situ*, but can be inferred from the genomic information. Community-level temperature-optima predictions showed that temperatures from the shallow sediments of BC12 (1–4 cm, average temperature of 27.28 °C) were significantly lower than in the deeper sediments (5–7 cm, average temperature of 30.65 °C, Supplementary Table S1). The community-level optimal temperatures for JL218P were close to those of the upper half of BC12. The genome-level optimal temperatures of *Hydrothermarchaeia*, the dominant class in the deeper layers (*n* = 7, with an average of 43.71 °C) were significantly higher than those of *Nitrososphaeria*, the dominant class from JL218P (*n* = 9, with an average of 32.93 °C).

**Figure 3:**
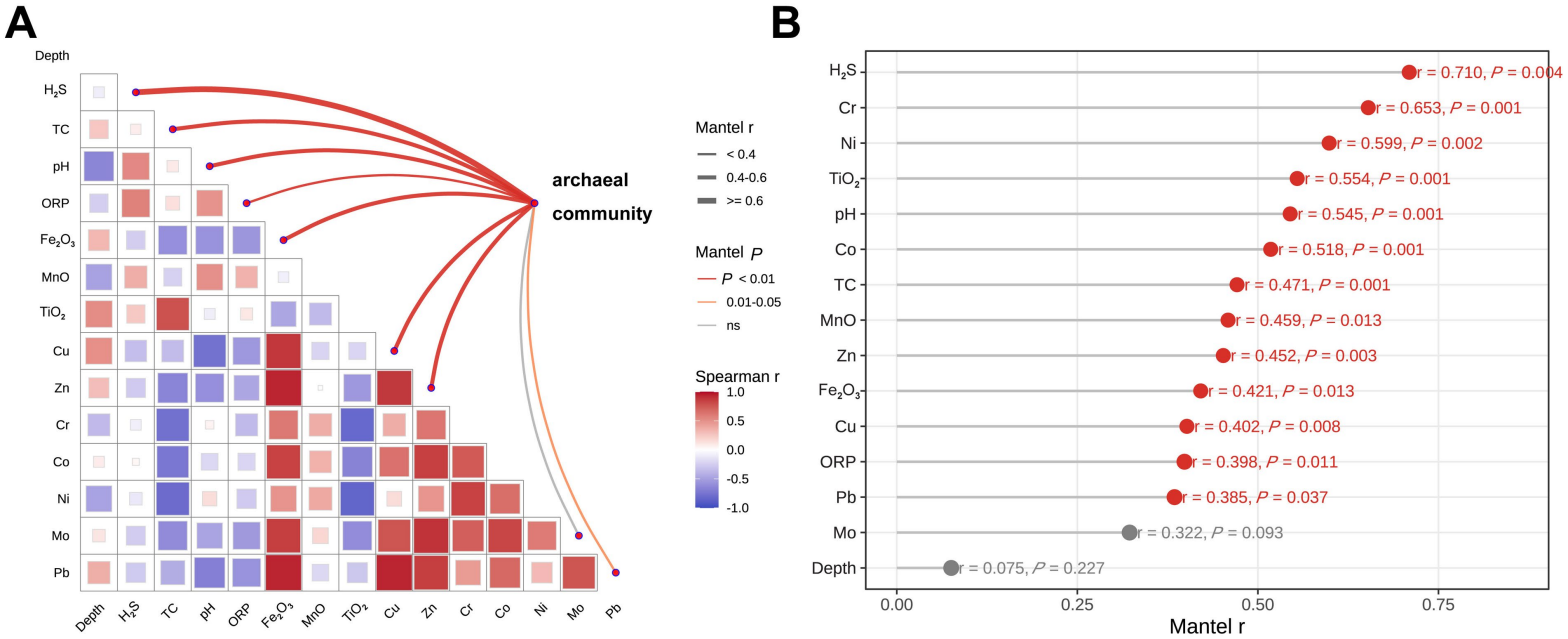
Correlations among geochemical variables and their associations with archaeal community structure. **(A)** Pairwise Spearman correlations among geochemical variables and Mantel test associations between individual environmental variables and archaeal community structure. In the lower triangular matrix, red and blue squares indicate positive and negative correlations, respectively, and square size reflects correlation strength. Curved lines represent Mantel test relationships between individual variables and archaeal community structure; line color denotes significance level and line width indicates Mantel’s *r* value. **(B)** Single-variable Mantel test results showing the correlation strength between each environmental factor and archaeal community structure. Red points indicate significant correlations (*p* < 0.05), whereas gray points indicate non-significant correlations. Values of Mantel’s r and *p* are shown for each variable.

As shown in Figure 3A, Mantel analysis revealed a strong overall relationship between the geochemical matrix and archaeal community structure across all subsamples (Mantel’s *r* = 0.752, *p* = 0.001; FDR = 0.001). Pairwise Spearman correlations further showed substantial covariation among environmental variables. Single-variable Mantel tests (Figure 3B) showed that 13 of the selected environmental variables were significantly correlated with archaeal community structure. The strongest associations were detected for H_2_S, Cr, Ni, TiO_2_, pH, and Co, whereas moderate but significant correlations were also observed for TC, Zn, Cu, ORP, Fe_2_O_3_, MnO, and Pb. In contrast, depth was not significantly associated with variation in the archaeal community, and Mo also showed no significant effect. Together, these results indicate that archaeal community turnover was more closely linked to geochemical gradients rather than to sediment depth itself.

**Figure 4:**
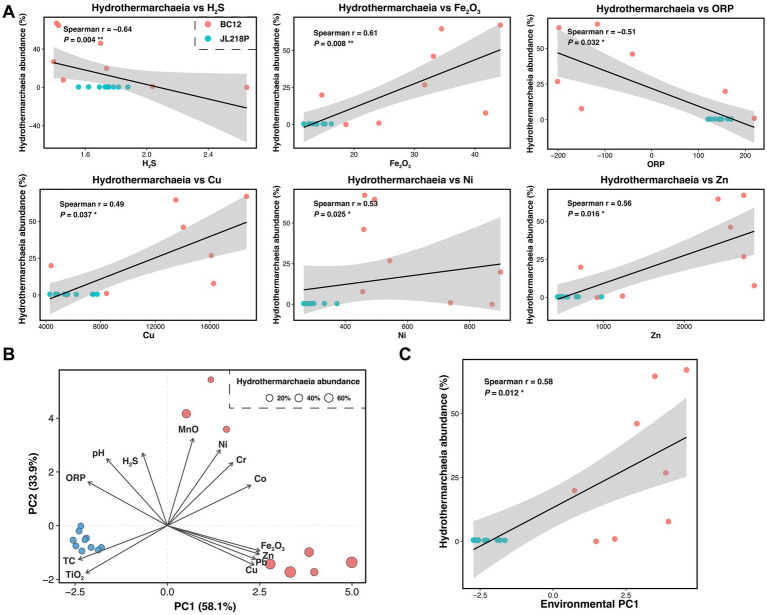
Associations of *Hydrothermarchaeia* abundance with environmental variables and geochemical gradients. **(A)** Scatterplots showing the associations between *Hydrothermarchaeia* relative abundance and selected environmental variables (H_2_S, Fe_2_O_3_, ORP, Cu, Ni, and Zn). Points represent individual samples and are colored by site. **(B)** PCA biplot of sediment geochemical variables. Samples point size represents *Hydrothermarchaeia* relative abundance. Arrows indicate the loadings of environmental variables on the first two principal components. **(C)** Scatterplot showing the association between *Hydrothermarchaeia* relative abundance and PC1 scores from the environmental PCA. Spearman’s correlation statistics are shown in the panel; the black line and gray shading indicate the fitted trend and 95% confidence interval, respectively.

To further resolve lineage-level responses to the observed geochemical gradients, we next examined the distribution of *Hydrothermarchaeia. Hydrothermarchaeia* abundance tracked sediment geochemical variation, showing negative correlations with H_2_S and ORP, but positive correlations with Fe_2_O_3_ and several trace metals (Figure 4A). PCA further identified a dominant environmental gradient contrasting Fe- and metal-enriched conditions with TC-, TiO_2_-, and ORP-associated sediments (Figure 4B), and *Hydrothermarchaeia* abundance increased significantly along this axis (Figure 4C). These patterns indicate that *Hydrothermarchaeia* is associated with the Fe- and metal-enriched end of the sediment geochemical gradient.

### 3.4 Archaeal co-occurrence networks across sediment cores

**Figure 5:**
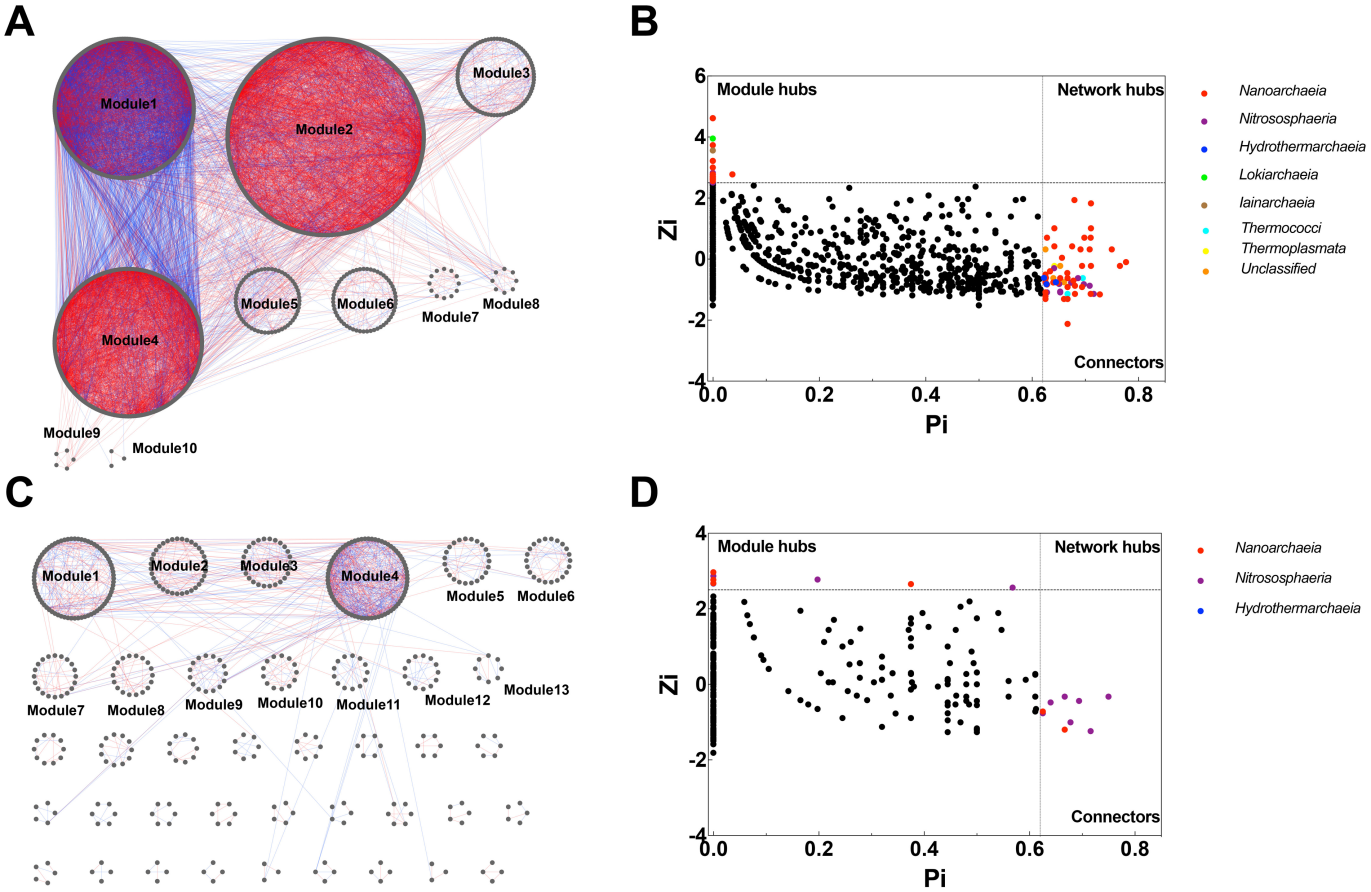
Co-occurrence network and Zi-Pi analysis of the archaeal microbiota in BC12 **(A,B)** and JL218P **(C,D)**. Each node indicates one OTU. Red edge indicates a positive interaction, while blue edge indicates a negative interaction between two nodes.

Co-occurrence network analysis revealed marked differences in the structural characteristics of the archaeal network between the two sediment cores (Figures 5A,C). The BC12 network (1,351 nodes and 15,345 edges) exhibited greater complexity and a larger size than the JL218P network (724 nodes and 1,296 edges). Class-level composition also differed between the two networks. In BC12, the network was dominated by *Nanoarchaeia* (~65.43%), followed by *Nitrososphaeria* (~18.80%), *Hydrothermarchaeia* (~3.18%), *Thermoplasmata* (1.85%), and *Bathyarchaeia* (1.63%). In contrast, JL218P showed a more balanced composition between *Nanoarchaeia* (~52.35%) and *Nitrososphaeria* (~35.50%), with substantially low representation of other archaeal groups (Supplementary Figures S3A,B). These results indicate pronounced differences in archaeal network composition between sediment located at different positions along the hydrothermal gradient.

Descriptive comparison of key topological properties showed consistently higher values in BC12 than in JL218P, including average clustering coefficient (0.398), density (0.017), centralization of betweenness (0.029), connectedness (0.984), average degree (22.717), and the proportion of positive interactions (68.17%) (Table 2). In addition, the distributions of node-level degree and local clustering coefficient differed significantly between the two networks (Supplementary Figures S3C,D). Overall, these features suggest a more highly connected archaeal network in the near-vent sediments, with stronger local clustering and a greater potential for cooperative interactions.

**Table 2:** Topological properties of archaeal community co-occurrence networks in sediments.

Network type	Network indexes	BC12	JL218P
Empirical networks	Total nodes	1,351	724
	Total links	15,345	1,296
	*R*^2^ of power-law	0.673	0.989
	Average degree (avgK)	22.717	3.580
	Average clustering coefficient (avgCC)	0.398	0.168
	Density (D)	0.017	0.005
	Average path length (GD)	4.151	6.415
	Harmonic geodesic distance (HD)	3.625	4.93
	Modularity	0.546	0.683
	Centralization of betweenness (CB)	0.029	0.026
	Connectedness (Con)	0.984	0.284
	Positive interaction	68.17%	54.24%
	Positive/negative	2.142	1.185
Random networks	Harmonic geodesic distance (HD)	2.555 ± 0.004	3.825 ± 0.036
	Average clustering coefficient (avgCC)	0.009 ± 0.002	0.001 ± 0.001
	Modularity	0.157 ± 0.002	0.534 ± 0.005

Zi-Pi analysis further revealed clear differences in the composition and distribution of keystone taxa between the two sites (Figures 5B,D). The BC12 network contained 99 keystone taxa, representing 7.33% of all nodes, including 66 connectors and 33 module hubs. These keystone taxa were affiliated with several archaeal lineages, particularly *Nanoarchaeia*, *Nitrososphaeria*, and *Hydrothermarchaeia*. In contrast, only 17 keystone taxa (2.35% of nodes) were identified in JL218P, and these were mainly assigned to *Nitrososphaeria* and *Nanoarchaeia*. Together, these results show that archaeal co-occurrence patterns differed substantially between the two sediment cores, with the near-vent site exhibiting a larger, more connected, and taxonomically broader network than the far-vent site.

### 3.5 Genomic features of *Hydrothermarchaeia* MAGs in BC12 sediments

Metagenomic binning recovered 282 MAGs meeting the quality criteria from BC12 sediments, including 22 archaeal MAGs. The taxonomic assignment of all qualified archaeal genomes was provided in Supplementary Table S2. Among these archaeal MAGs, we paid particular attention to *Hydrothermarchaeia* because community composition analysis showed that this group increased significantly in relative abundance in the deeper BC12 sediments. Seven MAGs were classified as *Hydrothermarchaeia*, accounting for 31.82% of the recovered archaeal MAGs. These *Hydrothermarchaeia* MAGs showed estimated completeness >70% and contamination <10% (Table 3).

**Table 3:** Genomic information of *Hydrothermarchaeia* MAGs.

MAG	Source bin ID	Size (bp)	Completeness (%)	Contamination (%)	GC	N50	Abundance
MAG1	BC1204_bin3	1,363,691	76.16	2.552	0.481	5,414	1.595327
MAG2	BC1206_bin6	1,586,314	96.26	1.869	0.486	72,733	5.16859
MAG3	BC1206_bin13	1,628,188	87.85	0.0	0.478	65,850	5.97403
MAG4	BC1206_bin55	1,277,760	80.06	0.934	0.48	7,167	8.187897
MAG5	BC1207_bin6	2,424,293	96.72	6.542	0.473	81,838	10.722834
MAG6	BC1207_bin17	1,364,744	71.02	1.006	0.477	7,077	1.088474
MAG7	BC1207_bin19	1,118,533	81.30	0.0	0.484	10,011	8.233094

**Figure 6:**
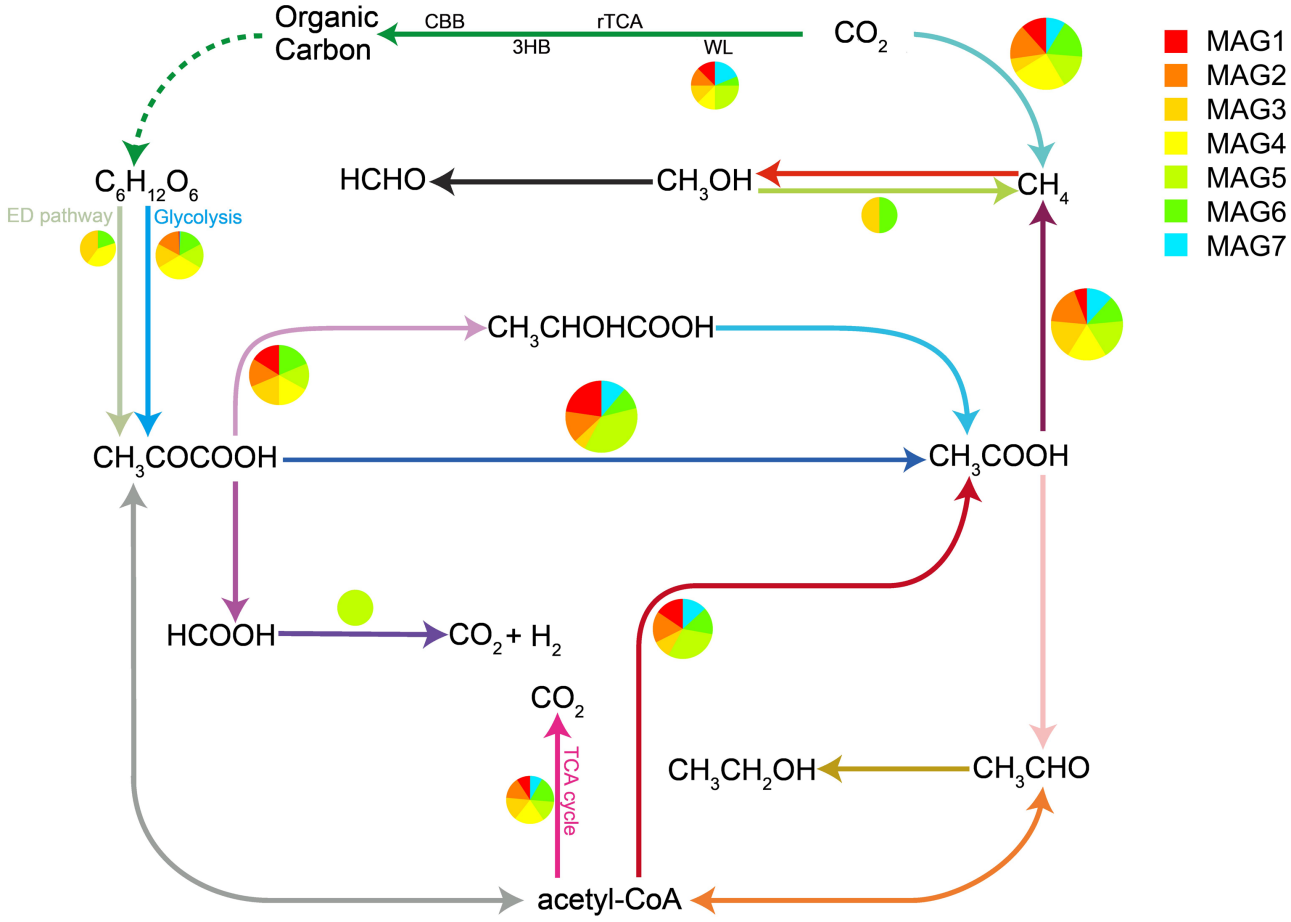
Reconstructed carbon metabolic network of *Hydrothermarchaeia* MAGs from BC12 sediment based on metagenomic annotations. Pathways were inferred from annotated key genes in the recovered MAGs. Pie charts indicate the relative abundance of pathway-associated genes across metagenomic samples.

KEGG-based functional annotation revealed that these *Hydrothermarchaeia* MAGs encoded genes involved in energy conservation, carbon metabolism, and environmental sensing (Supplementary Table S3). Reconstruction of carbon metabolic pathways indicated the presence of genes associated with the Wood-Ljungdahl (WL) pathway, glycolysis, the Entner-Doudoroff (ED) pathway, and a partial tricarboxylic acid (TCA) cycle, suggesting a potential for carbon transformation and assimilation. The annotated genes further indicated a potential capacity for the utilization and interconversion of simple organic substrates, particularly sugars, acetate, and formate, with pyruvate and acetyl-CoA serving as central nodes in low-molecular-weight organic matter turnover (Figure 6). In addition, genes associated with anaerobic respiration using terminal electron acceptors, particularly nitrate and sulfate, were also identified. In contrast, genes encoding key components of canonical aerobic respiration were not detected in the analyzed MAGs, suggesting that *Hydrothermarchaeia* in BC12 sediments likely adopt an anaerobic or oxygen-limited lifestyle. Functional annotation also identified the bacterial chemotaxis pathway, including core genes such as *mcp*, *che*A, *che*W, *che*R, *che*B, *che*Y, and *che*Z, indicating a potential capacity for chemotactic sensing and response to environmental gradients.

### 3.6 Functional prediction of sedimentary archaeal communities

**Figure 7:**
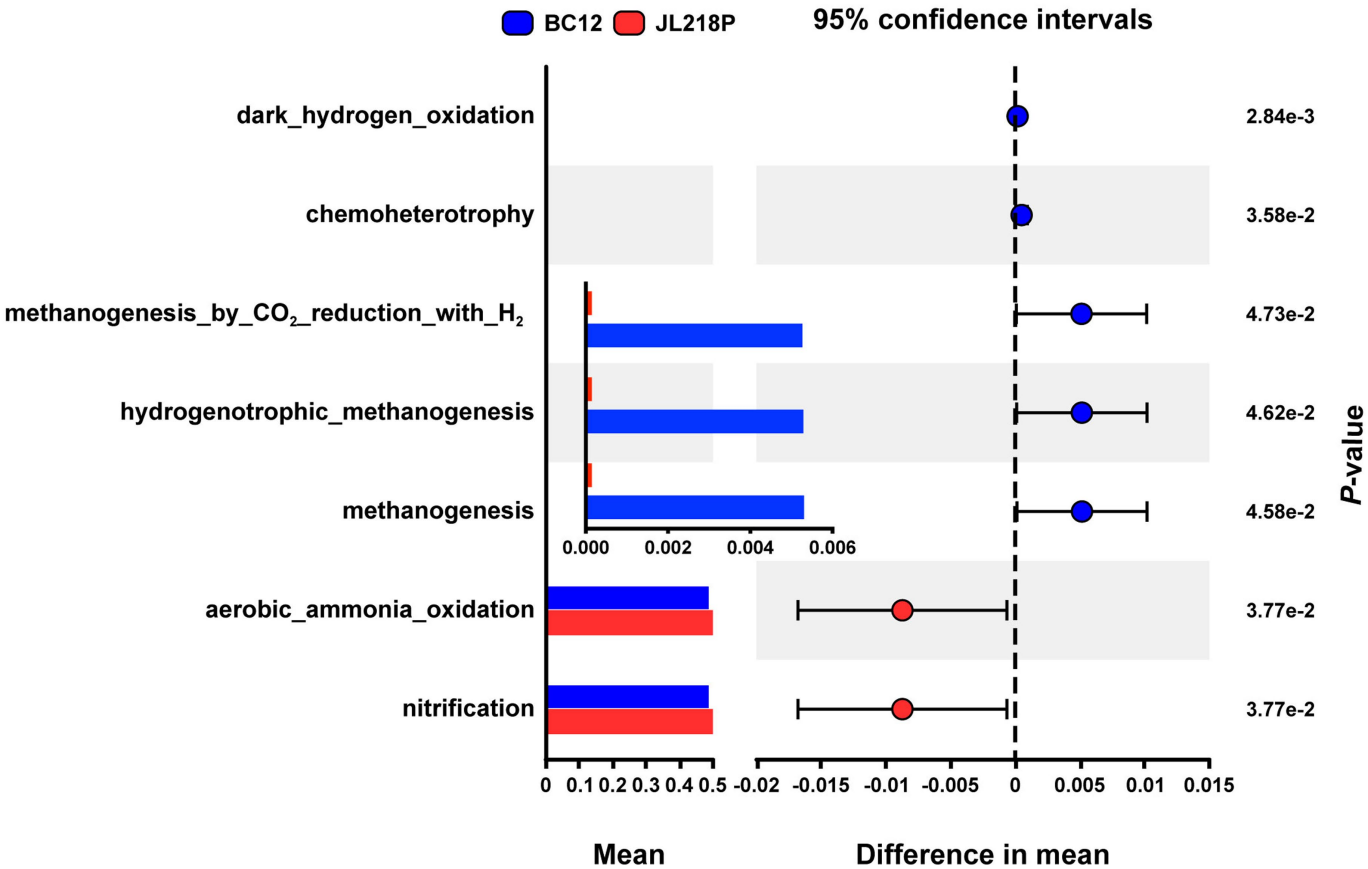
STAMP analyses of FAPROTAX functional predictions. The bar chart shows the average proportion of each metabolic function, and the error plot displays the 95% confidence intervals.

Functional prediction based on FAPROTAX identified seven metabolic functions that differed significantly between the two sediment cores (*p* < 0.05) (Figure 7). Among these, BC12 showed higher abundances of methanogenesis, hydrogenotrophic methanogenesis, methanogenesis by CO_2_ reduction with H_2_, chemoheterotrophy, and dark hydrogen oxidation, whereas JL218P showed high relative abundances in nitrification and aerobic ammonia oxidation. The composition and vertical distribution of these predicted functions differed clearly between the two sites.

JL218P was characterized by consistently higher relative abundance of nitrification and aerobic ammonia oxidation throughout the sediment profile. In contrast, BC12 showed higher relative abundances of methane-related functions. In particular, hydrogenotrophic methanogenesis and methanogenesis by CO_2_ reduction with H_2_ were significantly more abundant in BC12 than in JL218P. These methanogenesis-related functions also displayed clear vertical variation, with relative abundances increasing in the deeper sediment layers (5–8 cm) of BC12. In addition, dark hydrogen oxidation was significantly enriched in BC12.

To further evaluate the FAPROTAX-based functional predictions, we compared them with metagenomic gene annotations for key methane- and nitrogen-cycling pathways (Supplementary Figure S4). Methanogenesis-related genes, including *mcrA*, *mcrB*, *mcrG*, and *mtrA*-*mtrH*, were more abundant in BC12, whereas nitrification-related genes, including *amoA*, *amoB*, *amoC*, *hao*, *nxrA*, and *nxrB*, were relatively more abundant in JL218P. In addition, several genes related to protein folding, damage repair, and cellular protection were enriched in the deeper BC12 samples, including *groEL*, *clpB*, *grpE*, *hsp20*, trehalose-related functions, and thermosome (Supplementary Figure S5).

## 4 Discussion

### 4.1 Hydrothermal inputs shape archaeal community structure and indicator taxa

Our results demonstrated that hydrothermal-related geochemical heterogeneity is a major factor structuring archaeal communities in the sediments of the Tianxiu hydrothermal field. Archaeal assemblages differed markedly between the strongly hydrothermally influenced core BC12 and the more distal core JL218P, consistent with strong environmental filtering under contrasting geochemical regimes. Notably, the enrichment of *Hydrothermarchaeota* in the deeper layers of BC12 may reflect a legacy of stronger hydrothermal influence in these sediments, in accordance with higher community-level optimal temperature predictions (Supplementary Table S1). This depth-associated pattern further suggests that past hydrothermal fluid activity may have left a persistent record on archaeal community composition and diversity.

Among the taxa enriched in BC12, *Hydrothermarchaeia* were particularly notable in both the co-occurrence network and community composition. This lineage has previously been reported from hydrothermal deposits and other energy-rich anoxic environments, where it has been implicated in multiple biogeochemical processes (Carr et al., 2019; Zhou et al., 2020). In our dataset, Hydrothermarchaeia abundance covaried with hydrothermal-related geochemical gradients, showing negative associations with H_2_S and ORP and positive associations with Fe_2_O_3_ and several trace metals. This pattern, together with the positive relationship between *Hydrothermarchaeia* abundance and environmental PC1, links this lineage to the Fe- and metal-enriched end of the sediment geochemical spectrum. Genomic annotation of our MAGs further identified genes affiliated with the Wood-Ljungdahl pathway, suggesting the potential for carbon assimilation via the reductive acetyl-CoA pathway. Given that this pathway is among the most energy-efficient carbon fixation strategies known, with minimal ATP requirement (Berg et al., 2010; Bar-Even et al., 2012), its occurrence may reflect adaptation to the reducing and energetically constrained conditions typical of hydrothermally influenced subsurface sediments. These features are consistent with a possible role for *Hydrothermarchaeia* in *in situ* carbon cycling. In addition, previous genomic studies have suggested that members of this lineage may possess capacities for carboxydotrophy, anaerobic respiration, and flexible substrate utilization in hydrothermal systems (Carr et al., 2019). The detection of chemotaxis-related genes in our dataset further indicates that these organisms may be capable of sensing and responding to steep geochemical gradients, which are characteristic of hydrothermal sediments. Taken together, our results highlight *Hydrothermarchaeia* as a potentially important archaeal lineage in BC12, with possible involvement in carbon cycling and other redox-linked processes in hydrothermally influenced sediments.

The distribution pattern of *Hydrothermarchaeia* also supports its potential value as a hydrothermal-associated indicator lineage. Previous work has reported an increased abundance of *Hydrothermarchaeia* from the outer to inner portions of inactive hydrothermal chimneys, pointing to a close ecological association with hydrothermal mineral structures and chemically reduced habitats (Suzuki et al., 2004). In our study, the marked enrichment in the deeper BC12 layers (5–8 cm) is consistent with this interpretation and may reflect stronger historical exposure to hydrothermal inputs. Although additional sedimentological and temporal evidence would be required to confirm the timing and intensity of such events, the observed vertical pattern supports the idea that *Hydrothermarchaeota* respond sensitively to hydrothermal forcing and may persist as a biological signature of past fluid influence.

Besides *Hydrothermarchaeota*, BC12 was also enriched in *Nanoarchaeia* and *Thermoplasmata* relative to JL218P. Both archaeal groups have been repeatedly reported from hydrothermal systems and other extreme marine habitats (Wurch et al., 2016; St. John et al., 2019). *Nanoarchaeia* are known for their host-associated or symbiotic lifestyles and are often linked to tightly coupled metabolic interactions in thermally active environments. Their enrichment at BC12 may therefore reflect a community with stronger interdependence and niche specialization under hydrothermal conditions. *Thermoplasmata*, many members of which are thermophilic or acidophilic, have been associated with sulfur and iron transformations in geothermal and hydrothermal settings (Reysenbach et al., 2006). The co-occurrence of these archaeal groups with *Hydrothermarchaeota* further supports the interpretation that hydrothermal input strongly selects for lineages adapted to reduced, dynamic, and chemically heterogeneous habitats.

In contrast, the archaeal community in JL218P was dominated by *Nitrososphaeria* and remained comparatively stable along the sediment depths. This pattern suggests a more stable depositional setting with weaker hydrothermal disturbance. Members of *Nitrososphaeria* are widely recognized as key ammonia-oxidizing archaea and are typically favored in relatively oxidized environments where nitrification can be sustained (Trouche et al., 2023; Wright and Lehtovirta-Morley, 2023). Their predominance in JL218P is therefore consistent with the higher abundance of predicted nitrification and aerobic ammonia oxidation functions observed in this core. By comparison, the lower abundance of *Nitrososphaeria* in the deeper BC12 sediments likely reflects the more reduced conditions associated with hydrothermal influence. These contrasting distributions highlight that the sediment redox conditions shape the distribution of archaeal groups adapted to hydrothermal settings.

Comparison with previous studies suggests that hydrothermal archaeal communities are commonly dominated by major lineages within *Crenarchaeota*, *Thaumarchaeota*, and *Euryarchaeota*, despite strong site-specific variation in taxonomic composition and relative abundance (Zhang et al., 2015; Brandt and House, 2016; Zhang et al., 2016; Huang et al., 2023). Our results extend this view by showing that even within a single hydrothermal field, variation in hydrothermal influence can generate clear shifts in archaeal community structure, indicator taxa, and inferred ecological roles. Together, these findings suggest that hydrothermal input acts not only as a local geochemical driver but also as an important ecological filter shaping archaeal assembly and functional potential in deep-sea sediments.

### 4.2 Archaeal co-occurrence patterns along the hydrothermal gradient

The archaeal co-occurrence networks differed markedly between BC12 and JL218P, suggesting that variation in hydrothermal influence was associated with distinct patterns of community organization along the sediment gradient. Because BC12 experienced a stronger hydrothermal influence than JL218P, the contrasting network topologies likely reflect differences in local geochemical heterogeneity generated by hydrothermal forcing. This interpretation is consistent with ecological theory emphasizing the importance of environmental filtering in structuring microbial communities under contrasting physicochemical conditions (Barberán et al., 2012; Nemergut Diana et al., 2013; Kraft et al., 2015).

At BC12, the larger network size, higher connectivity, greater clustering, and broader phylogenetic distribution of putative keystone taxa indicate a more tightly structured archaeal assemblage under stronger hydrothermal influence. Such a pattern is consistent with the strong microscale heterogeneity typical of hydrothermally influenced sediments, where steep gradients in temperature, redox conditions, and reduced compounds can expand niche availability and promote more extensive archaeal associations. The occurrence of both module hubs and connectors further indicates a more differentiated network organization, suggesting that multiple archaeal groups may link subnetworks distributed across distinct microhabitats. In this context, the predominance of *Nanoarchaeia* in the BC12 network may indicate the importance of host-associated or otherwise interdependent archaeal lineages in hydrothermally influenced sediments.

In contrast, the simpler network topology observed at JL218P suggests a comparatively restricted set of archaeal associations under weaker hydrothermal influence. Lower connectivity, few putative keystone taxa, and a narrower phylogenetic distribution of central nodes indicate that network organization at this site was centered on fewer archaeal lineages. The higher proportion of negative associations may reflect stronger niche partitioning or limited resource availability in these comparatively stable sediments (Faust and Raes, 2012). The concentration of keystone roles within *Nitrososphaeria* suggests that ammonia-oxidizing archaea may play a disproportionate role in structuring communities in the more homogeneous sediments under weaker hydrothermal influence.

Taken together, the network differences between BC12 and JL218P suggest that archaeal communities are organized in contrasting ways under hydrothermally influenced versus weakly influenced conditions. The more connected and phylogenetically broader network at BC12 is consistent with archaeal assemblages structured under stronger environmental heterogeneity, whereas the simpler architecture at JL218P appears to reflect a narrower range of ecological associations under comparatively weaker hydrothermal impact. Overall, these findings indicate that hydrothermal forcing influences archaeal communities at multiple levels, affecting not only taxonomic composition but also the topology of co-occurrence networks and the distribution of topologically important taxa in deep-sea sediments.

### 4.3 Environmental filtering of archaeal metabolic potential in hydrothermal sediments

Functional prediction further suggested that hydrothermal activity acts as a pivotal environmental driver shaping the archaeal metabolic potential along the sediment gradient. Consistent with the compositional and network differences observed between BC12 and JL218P, the inferred functional profiles indicated a shift from predominantly aerobic nitrogen cycling under weaker hydrothermal influence to more reduced-energy metabolisms under stronger hydrothermal influence. This pattern is ecologically plausible because hydrothermal inputs can strongly modify local redox conditions and substrate availability, thereby selecting for archaeal groups adapted to contrasting biogeochemical regimes.

At JL218P, which is less affected by hydrothermal activity, the higher relative abundance of predicted aerobic ammonia oxidation suggests that comparatively oxidizing and stable sediment conditions favored ammonia-oxidizing archaea. This pattern is consistent with the ecological preferences of *Nitrosopumilales* within the class *Nitrososphaeria* for oxic marine sediments, where they are major contributors to ammonia oxidation (Qin et al., 2017). Under such conditions, archaeal functional potential appears to be oriented more strongly toward nitrification-related processes, indicating that nitrogen turnover at JL218P may be supported primarily by aerobic chemolithotrophic pathways.

By contrast, the deeper sediments of BC12 showed a marked reduction in predicted aerobic ammonia oxidation together with enrichment of methanogenesis- and hydrogen-oxidation-related functions. Given the stronger hydrothermal influence at this site, this shift likely reflects selection imposed by more reducing conditions and greater availability of hydrothermal electron donors, particularly H_2_, CH_4_, and CO_2_, that are commonly associated with serpentinization-influenced systems (McCollom and Shock, 1997; Takai et al., 2008; Schrenk et al., 2013). Such conditions would be expected to constrain aerobic ammonia oxidation while favoring archaeal lineages capable of exploiting reduced substrates for anaerobic metabolism (Dang et al., 2010). The enrichment of predicted methanogenic potential is therefore consistent with the geochemical framework of hydrothermally altered sediments, where hydrogenotrophic methanogenesis may become increasingly competitive, particularly among lineages such as *Methanocaldococcus* spp. (Takai et al., 2008; Schrenk et al., 2013). Meanwhile, the low sulfate concentration in this environment likely mitigates competitive inhibition between sulfate-reducing bacteria and methanogens for substrates, thereby further promoting the relative enrichment of methanogenic functional groups (Oremland Ronald and Polcin, 1982). Likewise, the higher abundance of predicted dark hydrogen oxidation supports the idea that hydrogen-based energy metabolism represents an important functional feature of archaeal communities under stronger hydrothermal influence (Brazelton et al., 2010).

Although these patterns were inferred from FAPROTAX and should therefore be interpreted with caution, they provide only putative ecological indications based on taxonomic composition rather than direct evidence of metabolic function or activity. To avoid overinterpretation, we treat these results as hypothesis-generating patterns, while noting that metagenomic annotations of key methane- and nitrogen-cycling genes showed broadly consistent site-level trends, with relatively stronger methanogenesis-related potential in BC12 and stronger nitrification-related potential in JL218P. Metagenomic profiles from deeper BC12 sediments also showed enrichment of genes involved in protein folding, damage repair, and cellular protection, including chaperone- and stress response-related functions. These features suggest that microbial communities near the vent experience elevated physiological stress and require enhanced protein homeostasis under fluctuating and geochemically challenging conditions. Although TOME predictions did not show a strong overall increase in the community-level based metagenome-inferred environmental temperature across BC12, the deeper samples (5–7 cm) exhibited moderately higher predicted temperatures than the upper layers. Consistent with this pattern, several *Hydrothermarchaeia* MAGs recovered from this site displayed comparatively high predicted optimal growth temperature values (Table S1), supporting the interpretation that this lineage possesses thermal adaptation features consistent with a hydrothermal lifestyle.

Taken together, these results suggest that hydrothermal activity restructures archaeal metabolic potential by altering redox gradients, substrate availability, and physiological stress in deep-sea sediments. Under weaker hydrothermal influence, archaeal functional profiles were more closely associated with aerobic ammonia oxidation and nitrification, whereas stronger hydrothermal influence favored methanogenesis, hydrogen-dependent metabolism, and stress-tolerant lineages. In particular, the genomic features of *Hydrothermarchaeia* support the view that this archaeal group is adapted to hydrothermally influenced niches and may represent an important lineage in near-vent sediments.

## 5 Conclusion

Our study provides insight into how archaeal communities respond to contrasting hydrothermal influences in sediment cores from the Tianxiu hydrothermal field in the Northwest Indian Ocean. The results demonstrated a clear spatial divergence in archaeal community composition, diversity, co-occurrence patterns, and predicted metabolic potential between BC12 and JL218P, representing stronger and weaker hydrothermal influence, respectively. At the near-vent site BC12, sediments were enriched in *Hydrothermarchaeia* and formed a more complex co-occurrence network with a high proportion of positive associations. Functionally, this site showed enhanced predicted potential for methanogenesis and dark hydrogen oxidation, consistent with a more reduced sedimentary environment under stronger hydrothermal influence. In contrast, the far-vent site JL218P was dominated by *Nitrososphaeria*, exhibited a simpler network architecture with relatively more negative associations, and showed stronger predicted potential for nitrification and aerobic ammonia oxidation, suggesting archaeal functional profiles more closely linked to nitrogen cycling under comparatively more oxidizing conditions. Collectively, these findings highlight hydrothermal influence as a key environmental factor shaping archaeal community assembly, ecological organization, and functional differentiation in deep-sea sediments.

## Data Availability

The datasets presented in this study can be found in online repositories. The names of the repository/repositories and accession number(s) can be found at: https://www.ncbi.nlm.nih.gov/, PRJNA1441787.
