# Supplementary material for: Archaeal communities as indicators of hydrothermal influence in the Tianxiu vent field, Northwest Indian Ocean
Source: Front Microbiol. 2026 May 8;17:1837947. doi: 10.3389/fmicb.2026.1837947 (PMC13196343; doi:10.3389/fmicb.2026.1837947)
Supplement: Supplementary file 1 [file Supplementary_file_1.docx]

Supplementary Material

# Supplementary Figures


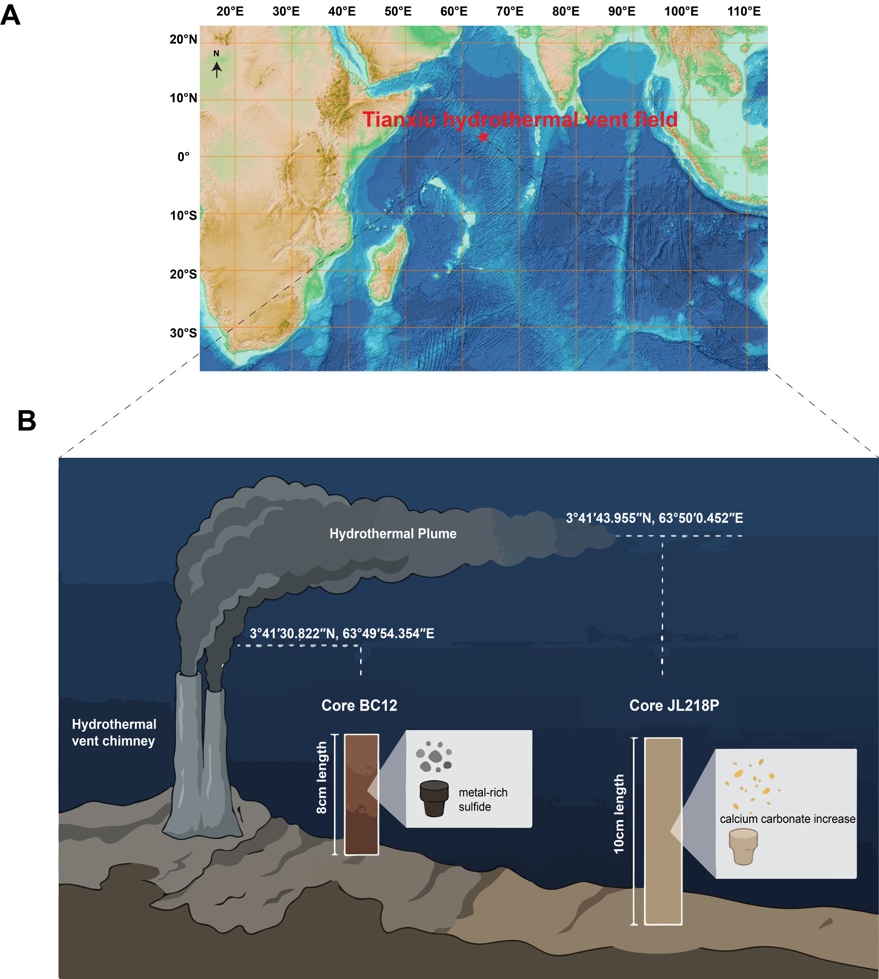


Supplementary Figure 1. Location of the study area.


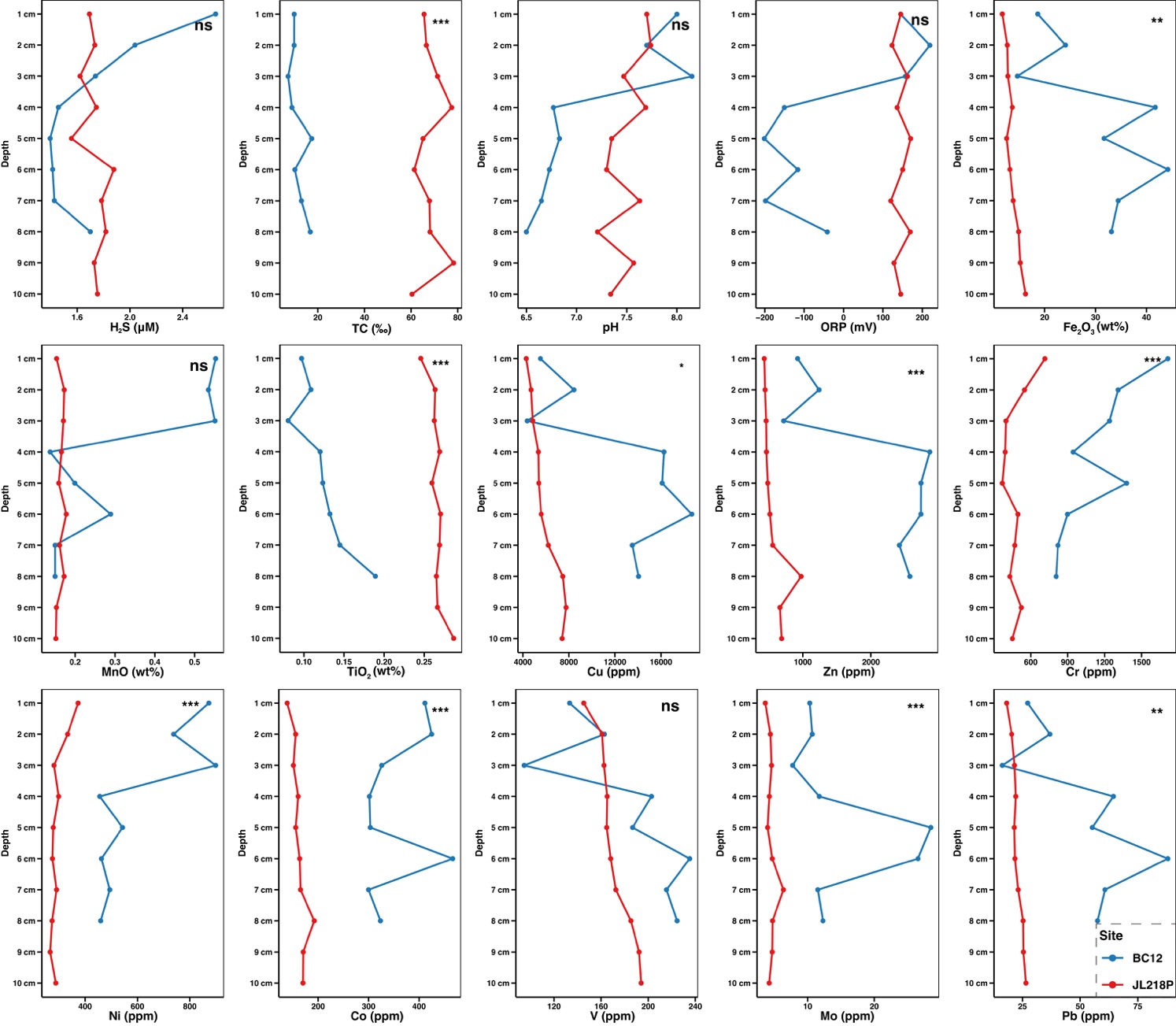


Supplementary Figure 2. Depth profiles of geochemical parameters in sediment cores BC12 and JL218P. Vertical variations in H_2_S, TC, pH, ORP, Fe_2_O_3_, MnO, TiO_2_, Cu, Zn, Cr, Ni, Co, V, Mo, and Pb were determined for both sediment cores to characterize site- and depth-dependent geochemical gradients. Blue lines represent BC12 and red lines represent JL218P. Statistical significance of differences between the two cores is indicated for each parameter (**P* < 0.05, ***P* < 0.01, ****P* < 0.001; ns, not significant).


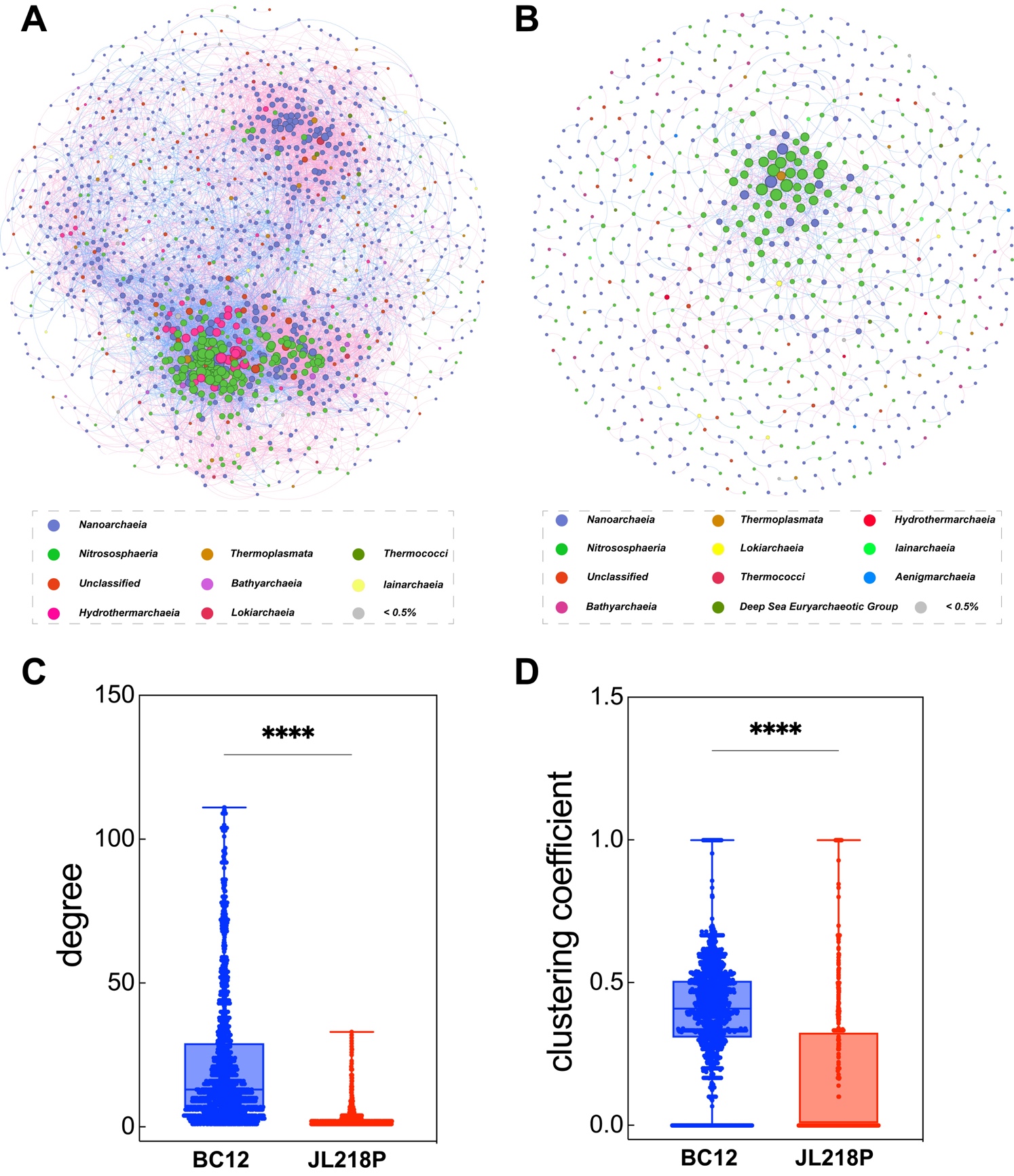


Supplementary Figure 3. Co-occurrence network of the archaeal communities in BC12 (A) and JL218P (B). Nodes were colored by class, and the node size was proportional to the number of connections. The red and blue edges represent positive/negative correlations, respectively. (C) Node degree (C) and clustering coefficient (D) distributions between the BC12 and JL218P networks. Differences in node-level degree and local clustering coefficient between the two networks were evaluated statistically; **** indicates *P* < 0.0001.


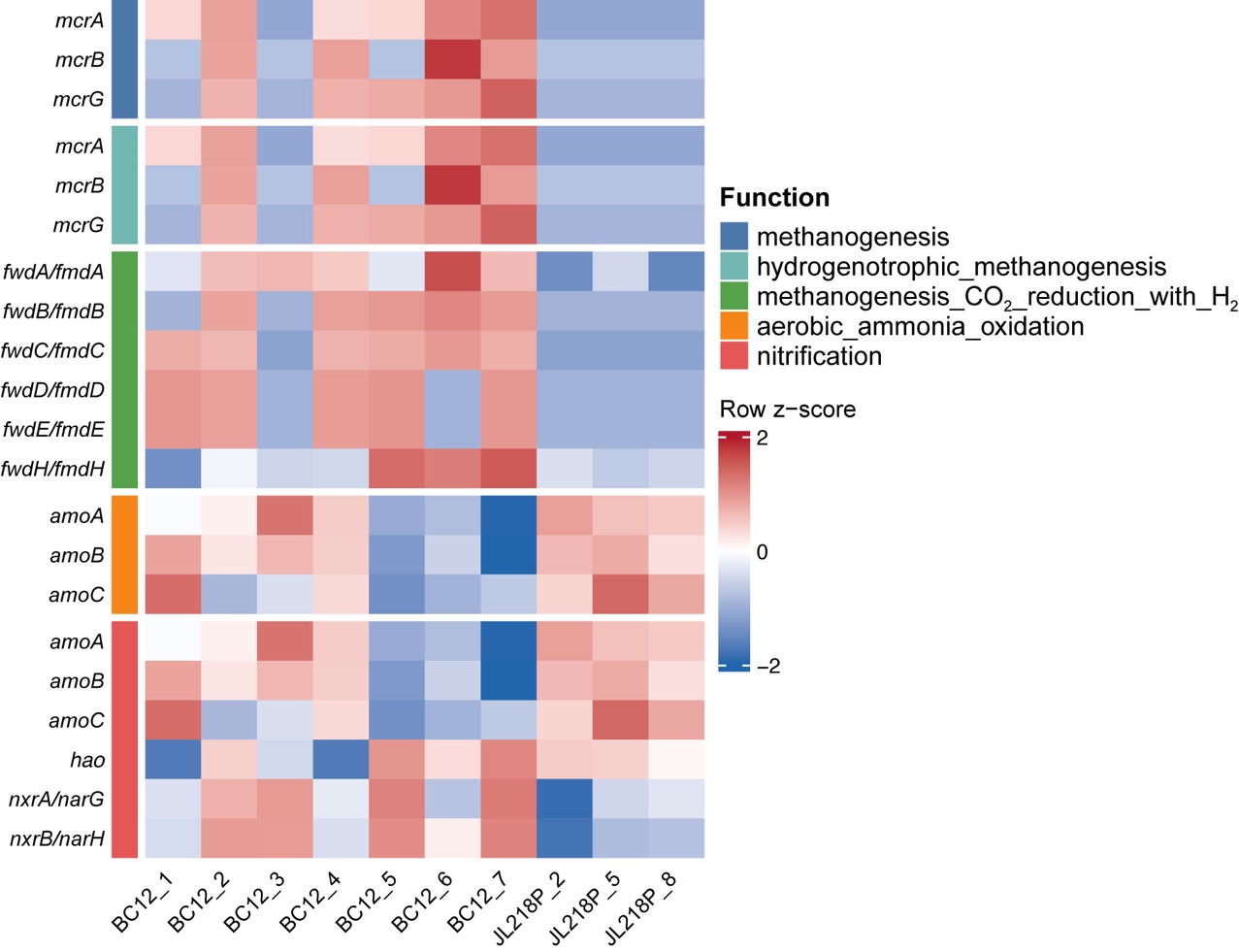


Supplementary Figure 4. Heatmap showing the distribution patterns of archaeal functional genes identified from metagenomic contigs across samples. Genes were grouped by predicted functions, including methanogenesis, hydrogenotrophic methanogenesis, aerobic ammonia oxidation, and nitrification.


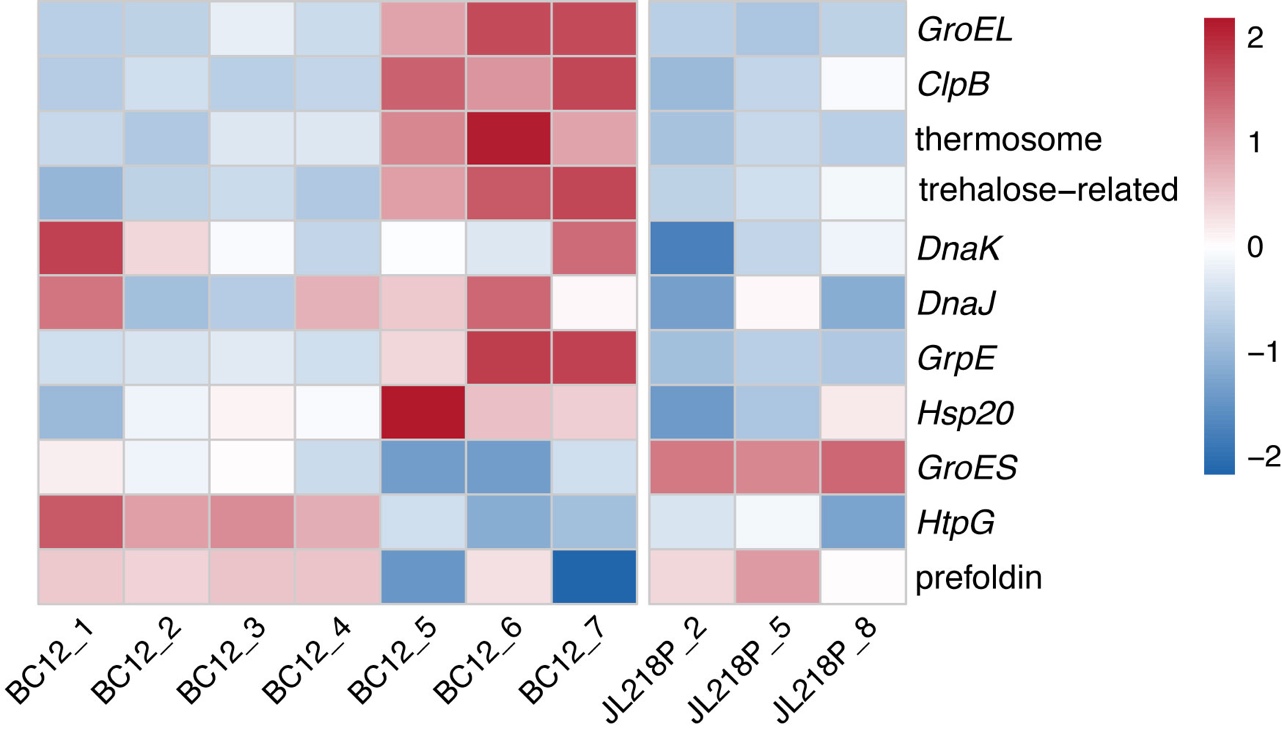


Supplementary Figure 5. Row-scaled heatmap of thermotolerance-related genes in BC12 and JL218P sediment samples. Colors represent row-wise z-scores calculated for each gene across all samples, with red indicating relatively higher and blue indicating relatively lower abundance within a given gene.

# Supplementary Tables

Supplementary Table 1. The predicted temperature for subsamples and individual MAGs

| **sample** | **predicted temperature (°C)** |
| --- | --- |
| BC12_1 | 27.07 |
| BC12_2 | 27.40 |
| BC12_3 | 27.40 |
| BC12_4 | 27.25 |
| BC12_5 | 30.45 |
| BC12_6 | 31.24 |
| BC12_7 | 30.27 |
| JL218P_2 | 27.98 |
| JL218P_5 | 28.42 |
| JL218P_8 | 28.32 |
| *Hydrothermarchaeia*_MAG1 | 45.78 |
| *Hydrothermarchaeia*_MAG2 | 40.48 |
| *Hydrothermarchaeia*_MAG3 | 48.51 |
| *Hydrothermarchaeia*_MAG4 | 35.87 |
| *Hydrothermarchaeia*_MAG5 | 41.66 |
| *Hydrothermarchaeia*_MAG6 | 48.57 |
| *Hydrothermarchaeia*_MAG7 | 45.07 |
| *BC12_Nitrososphaeria*_MAG1 | 27.38 |
| *BC12_Nitrososphaeria*_MAG2 | 28.73 |
| JL218P_*Nitrososphaeria*_MAG1 | 32.12 |
| JL218P_*Nitrososphaeria*_MAG2 | 34.55 |
| JL218P_*Nitrososphaeria*_MAG3 | 34.08 |
| JL218P_*Nitrososphaeria*_MAG4 | 35.59 |
| JL218P_*Nitrososphaeria*_MAG5 | 35.8 |
| JL218P_*Nitrososphaeria*_MAG6 | 34.09 |
| JL218P_*Nitrososphaeria*_MAG7 | 34.03 |

Supplementary Table 2. Taxonomic classification of all qualified archaeal MAGs recovered from BC12 sediments

| **MAGs** | **completeness** | **contamination** | **classification** |
| --- | --- | --- | --- |
| BC1202_bin29 | 96.73 | 1.6 | p_*Methanobacteriota*; c_*Methanopyri* |
| BC1203_bin3 | 75.32 | 2.987 | p_*Thermoproteota*; c_*Nitrososphaeria* |
| BC1204_bin23 | 77.36 | 1.733 | p_*Methanobacteriota*; c_*Methanopyri* |
| BC1204_bin3 | 76.16 | 2.552 | p_*Hydrothermarchaeota*; c_*Hydrothermarchaeia* |
| BC1205_bin24 | 75.93 | 1.2 | p_*Thermoplasmatota*; c_*Poseidoniia* |
| BC1205_bin47 | 85.51 | 0.0 | p_*Nanoarchaeota*; c_*Nanoarchaeia* |
| BC1205_bin5 | 78.52 | 3.271 | p_*Asgardarchaeota*; c_*Heimdallarchaeia* |
| BC1205_bin8 | 93.2 | 1.6 | p_*Thermoplasmatota*; c_*Thermoplasmata* |
| BC1206_bin13 | 87.85 | 0.0 | p_*Hydrothermarchaeota*; c_*Hydrothermarchaeia* |
| BC1206_bin21 | 85.51 | 0.934 | p_*EX4484-52*; c_*EX4484-52* |
| BC1206_bin3 | 91.14 | 2.4 | p_*Methanobacteriota*; c_*Methanopyri* |
| BC1206_bin50 | 89.95 | 2.180 | p_*Iainarchaeota*; c_*Iainarchaeia* |
| BC1206_bin52 | 88.47 | 3.271 | p_*Asgardarchaeota*; c_*Heimdallarchaeia* |
| BC1206_bin55 | 80.06 | 0.934 | p_*Hydrothermarchaeota*; c_*Hydrothermarchaeia* |
| BC1206_bin6 | 96.26 | 1.869 | p_*Hydrothermarchaeota*; c_*Hydrothermarchaeia* |
| BC1206_bin65 | 72.43 | 1.869 | p_*Iainarchaeota*; c_*Iainarchaeia* |
| BC1206_bin7 | 77.65 | 0.323 | p_*Thermoproteota*; c_*Nitrososphaeria* |
| BC1207_bin17 | 71.02 | 1.006 | p_*Hydrothermarchaeota*; c_*Hydrothermarchaeia* |
| BC1207_bin19 | 81.30 | 0.0 | p_*Hydrothermarchaeota*; c_*Hydrothermarchaeia* |
| BC1207_bin3 | 83.80 | 7.943 | p_*Asgardarchaeota*; c_*Heimdallarchaeia* |
| BC1207_bin34 | 91.93 | 1.6 | p_*Methanobacteriota*; c_*Methanopyri* |
| BC1207_bin6 | 96.72 | 6.542 | p_*Hydrothermarchaeota*; c_*Hydrothermarchaeia* |

Supplementary Table 3. Relative abundance of genes associated with selected non-carbon-related pathways in *Hydrothermarchaeia* MAGs from BC12 sediment.

| **pathway** | **MAG1** | **MAG2** | **MAG3** | **MAG4** | **MAG5** | **MAG6** | **MAG7** |
| --- | --- | --- | --- | --- | --- | --- | --- |
| Assimilatory sulfate reduction  (sulfate -> sulfite) | 6.00 | 6.42 | 29.17 | 29.17 | 22.00 | 19.17 | 0 |
| Dissimilatory sulfate reduction  (sulfate -> sulfite, *sat* and *apr*AB) | 18.25 | 26 | 49.75 | 49.25 | 33.75 | 49.75 | 12.5 |
| Dissimilatory sulfate reduction  (sulfite -> sulfide, *dsr*AB) | 0 | 25 | 0 | 0 | 25 | 0 | 25 |
| Dissimilatory nitrate reduction  (nitrite -> ammonia, *nir*BD or *nrf*AH) | 0 | 12 | 1 | 0 | 0.5 | 1 | 0 |
| Denitrification  (nitrous oxide -> nitrogen, *nos*Z) | 0 | 19 | 13 | 0 | 0 | 13 | 22 |
| Nitrogen fixation  (nitrogen -> ammonia, *nif*KDH) | 0 | 25 | 0 | 0 | 25 | 0 | 0 |
| chemotaxis | 1.04 | 0.04 | 9.69 | 9.73 | 0.12 | 4.92 | 1 |
